# Growth-coupled continuous directed evolution by MutaT7 enables efficient and automated enzyme engineering

**DOI:** 10.1128/aem.02491-24

**Published:** 2025-03-27

**Authors:** Yijie Deng, Kai Etheridge, Xinping Ran, Hannah E. Maurais, Rahul Sarpeshkar

**Affiliations:** 1 Thayer School of Engineering, Dartmouth College 145792, Hanover, New Hampshire, USA; 2 Department of Biochemical Engineering, Duke University 3065, Durham, North Carolina, USA; 3 Departments of Engineering, Microbiology & Immunology, Physics, and Molecular and Systems Biology, Dartmouth College 3728, Hanover, New Hampshire, USA; Kyoto University, Kyoto, Japan

**Keywords:** continuous evolution, MutaT7, *in vivo *mutagenesis, enzyme engineering, growth-coupled direction evolution

## Abstract

Traditional directed evolution is limited by labor-intensive iterative steps and low-throughput selection and screening. To address these challenges, we developed a growth-coupled continuous directed evolution (GCCDE) approach, enabling automated and efficient enzyme engineering. By linking enzyme activity to bacterial growth and utilizing the MutaT7 system, GCCDE combines *in vivo* mutagenesis and high-throughput selection of superior enzyme variants in a single process. To validate this approach, we evolved the thermostable enzyme CelB from *Pyrococcus furiosus* to enhance its β-galactosidase activity at lower temperatures while maintaining thermal stability. CelB activity was coupled to the growth of *E. coli*, allowing variants with improved activity to utilize lactose more efficiently and promote faster growth in a minimal medium. Using a continuous culture system, we achieved automated high-throughput mutagenesis and simultaneous real-time selection of over 10⁹ variants per culture. Integrating *in vitro* and *in vivo* mutagenesis further increased genetic diversity, yielding CelB variants with significantly enhanced low-temperature activity compared to the wild type while preserving thermostability. DNA sequencing identified key mutations likely responsible for improved substrate binding and catalytic turnover. This GCCDE approach is broadly applicable for optimizing diverse enzymes, demonstrating the potential of automated continuous evolution for industrial and research applications.

**IMPORTANCE:**

Enzyme engineering aims to develop enzymes with improved or novel traits, but traditional methods are slow and require repetitive manual steps. This study presents a faster, automated protein engineering approach. We utilized an *in vivo* mutagenesis technique, MutaT7 tools, to induce mutations in living bacteria and established a direct link between enzyme activity and bacterial growth. A continuous culture setup enables automated mutagenesis and growth-coupled selection of better-performing variants in real time. Bacteria with improved enzymes grew faster, selecting superior variants without manual intervention. Using this method, we engineered CelB with better performance at lower temperatures while maintaining thermal stability. By combining high-throughput mutagenesis and selection in a single process, this system bypasses iterative cycles of error-prone PCR, transformation, and screening. Our approach is adaptable to various enzymes, providing a faster and more efficient solution for enzyme engineering.

## INTRODUCTION

Directed evolution mimics natural evolutionary processes in the laboratory and has become a powerful tool for engineering proteins and regulatory elements, with applications spanning therapeutics, diagnostics, and metabolic engineering (1, 2). By introducing genetic diversity and selecting advantageous phenotypes, this technique rapidly enhances the desired performance of enzymes and other proteins. However, traditional directed evolution relies on iterative rounds of *in vitro* mutagenesis, transformation, and selection or screening, which are labor-intensive, time-consuming, and limit efficiency and scalability in protein engineering (2–4).

To overcome these limitations, *in vivo* directed evolution methods have emerged, enabling continuous evolution of target proteins within living systems (3–5). Techniques such as phage-assisted continuous evolution (PACE) (6), orthogonal DNA Replication (OrthoRep) (7), and MutaT7 (8) have significantly advanced the field of protein engineering. Among these, the MutaT7 system uses a hypermutator chimera protein, consisting of T7 RNA polymerase fused to a cytidine deaminase, to efficiently generate mutations in bacterial cells (8). This system introduces C-to-T or G-to-A mutations in regions downstream of the T7 promoter. Given its simplicity and effectiveness, enhanced variants of MutaT7 have been developed to induce all possible transition mutations, further expanding its utility (9–11). Compared to in *vitro* methods, MutaT7 bypasses iterative steps like error-prone PCR and cloning, greatly accelerating the process of protein engineering (8–10).

Although *in vivo* mutagenesis has promoted the efficiency of directed evolution, high-throughput selection remains a major bottleneck. Growth-coupled directed evolution addresses this challenge by linking enzymatic activity to bacterial growth under selective conditions. Variants with enhanced activity promote faster growth, leading to their enrichment, while less functional variants are outcompeted in the cell population. Growth coupling can be achieved through several strategies, including enabling the targeted protein to complement auxotrophic deficiencies, to detoxify harmful compounds, or to regulate reporter gene expression linked to cell growth (12). These strategies enable the high-throughput selection of enzyme variants *in vivo* and facilitate automation (5, 12).

In this study, we developed a growth-coupled continuous directed evolution (GCCDE) system that combines MutaT7 mutagenesis with a continuous culture technique to enable efficient enzyme evolution. By integrating automated mutagenesis, selection, and enrichment within a continuous culture system, GCCDE eliminates the need for iterative cycles of error-prone PCR, transformation, and screening, significantly reducing both time and effort. Our approach facilitates rapid evolution of large variant libraries while allowing precisely tuning selective pressures over extended periods. To demonstrate its effectiveness, we applied GCCDE to engineer the thermostable enzyme CelB from *Pyrococcus furiosus* (13, 14), a tetrameric protein with both β-glucosidase and β-galactosidase activities at high temperatures (15). Our goal was to enhance CelB activity at lower temperatures while retaining its thermostability. Using GCCDE, we successfully evolved CelB variants with desired properties in a single evolution experiment. This adaptable method underscores the potential of growth-coupled *in vivo* directed evolution for engineering a wide range of enzymes.

## RESULTS AND DISCUSSION

### Experimental design

**Fig 1:**
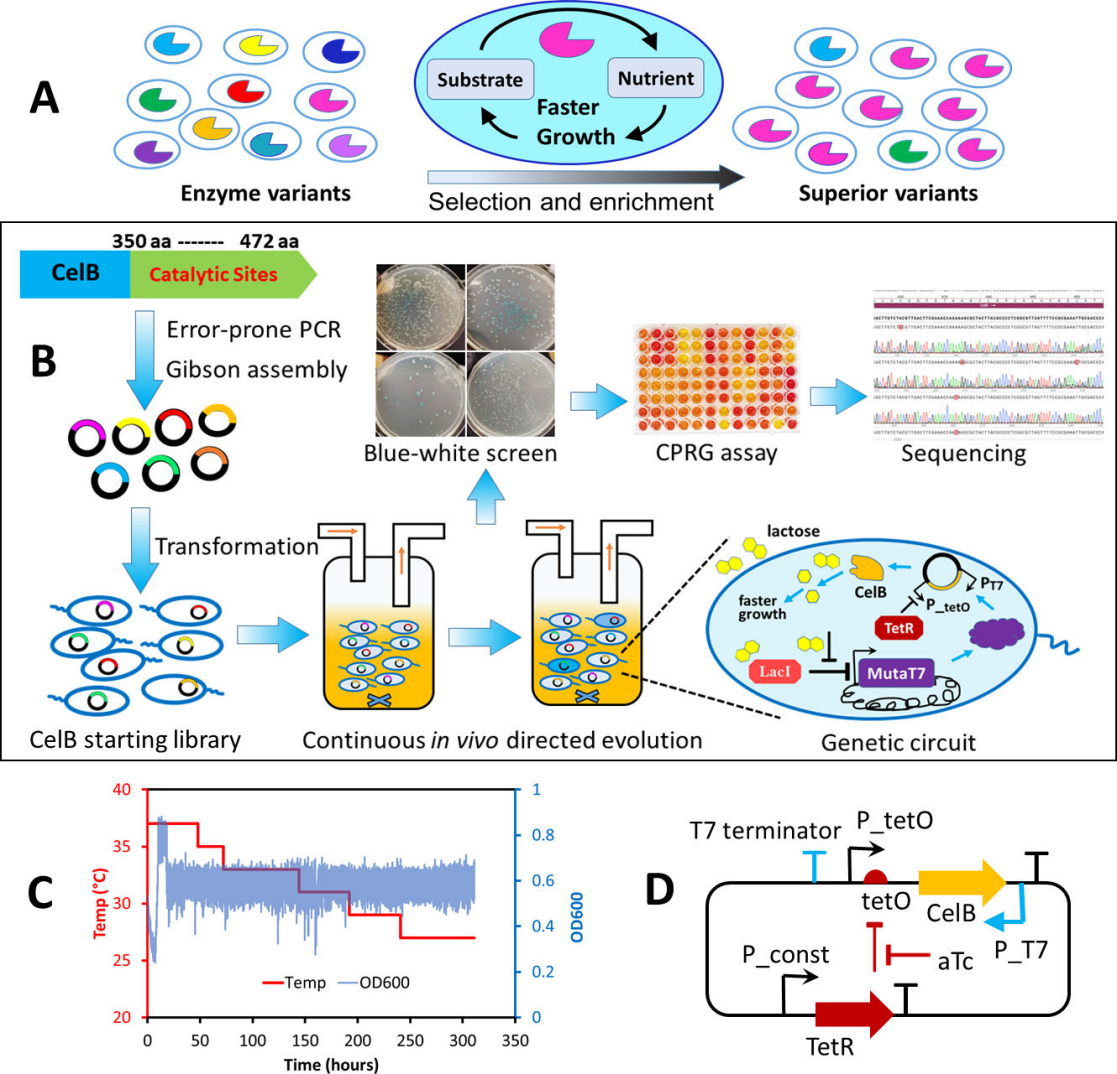
Experimental design of the growth-coupled *in vivo* continuous directed evolution. (A) Growth-coupling strategy to link enzymatic activity to cellular growth. (B) CelB starting library was generated via error-prone PCR (*in vitro* mutagenesis), followed by *in vivo* mutagenesis and high-throughput selection in a continuous culture system. Variants with enhanced activity were isolated from blue-white screening and confirmed by chlorophenol red-β-D-galactopyranoside (CPRG) assay prior to DNA sequencing. A simple genetic circuit was designed to enable flexible control of gene expression. Lactose in the medium binds to LacI, derepressing the expression of MutaT7, which is driven by the T7 promoter to generate mutations in the cell. CelB expression is induced by anhydrotetracycline (aTc) upon its binding to TetR. Continuous *in vivo* directed evolution selects superior variants that promote faster growth. (C) The culture temperature was gradually reduced from 37°C to 27°C to select CelB mutants with higher activity at lower temperatures. The cell density was maintained at an OD_600_ of approximately 0.6 during the exponential phase in a turbidostat device. (D) The low-copy-number plasmid designed for this study, carrying CelB, served as both the template for error-prone PCR and the vehicle for *in vivo* mutagenesis. CelB expression is regulated by TetR under the control of a hybrid promoter (P_tetO), which is modified from the T5 promoter by incorporating *tetO*.

A critical step in growth-coupled *in vivo* directed evolution is establishing a direct link between bacterial growth and the activity of a targeted enzyme. This requires a host bacterial strain lacking the native activity of the target enzyme, ensuring that bacterial growth in a defined medium depends entirely on the activity of the enzyme introduced via a plasmid. The growth medium must include the enzyme’s substrate, which is converted into essential nutrients for bacterial proliferation. Variants with higher enzymatic activity utilize the substrate more efficiently, promoting faster growth and enrichment of superior variants over time (Fig. 1A).

As a demonstration, we targeted the thermostable CelB protein (13, 14) to improve its β-galactosidase activity at lower temperatures while maintaining its stability at high temperatures. CelB activity was coupled to bacterial growth by using *E. coli* Dual7 strain (10) as the host. This strain, derived from DH10B, contains mutations in the *lacZ* gene, rendering its β-galactosidase activity negligible. The Dual7 strain also integrates MutaT7 proteins into its chromosome, which can be induced by lactose or isopropyl β-D-1-thiogalactopyranoside (IPTG). Additionally, the Dual7 strain carries a Δ*ung* mutation to enhance *in vivo* mutagenesis efficiency by preventing the repair of induced mutations (8, 10). This strain, transformed with a plasmid library of CelB, was cultivated in a lactose minimal medium where lactose served as the sole carbon source. Enhanced CelB variants converted lactose into glucose and galactose more efficiently, enabling faster bacterial replication. Cells with less effective variants grew slower and were gradually washed out in the continuous culture system, leading to the enrichment of highly active CelB variants (Fig. 1B). Selective pressure was applied by gradually lowering the culture temperature from 37°C to 27°C, favoring the evolution of CelB variants with improved activity at lower temperatures (Fig. 1C).

We designed a simple genetic circuit to allow for flexible gene regulation. A low-copy-number plasmid carrying the *celB* gene under the control of a hybrid promoter P_tetO allowed CelB expression to be regulated by the external inducer anhydrotetracycline (aTc) (Fig. 1D). Lactose in the medium activated the expression of MutaT7 proteins, initiating *in vivo* mutagenesis. A T7 promoter downstream of the *celB* gene drove evolution in a different direction, ensuring independent regulation of mutagenesis and CelB expression, while a T7 terminator upstream of the hybrid promoter P_tetO minimized off-target mutations (Fig. 1D). Interestingly, the system inherently includes a beneficial negative feedback loop. As CelB activity increases and more lactose is metabolized, the reduced availability of lactose for MutaT7 expression naturally slows down mutation rates. This mechanism could help stabilize the system when the targeted protein approaches its optimal function. Although this feedback loop was not a significant factor in our current experiment (due to the high lactose concentration used in the medium saturating the LacI protein), it holds potential for enhancing future circuit designs in *in vivo* directed evolution.

To address the limitations of MutaT7, which introduces only transition mutations, we used error-prone PCR to generate a diverse set of mutations in the starting library (Fig. 1B) from the above CelB plasmid (Fig. 1D) before initiating *in vivo* mutagenesis. The combination of *in vitro* and *in vivo* mutagenesis could expand the genetic diversity of CelB and improve the system’s evolutionary outcomes (Fig. 1B).

By integrating mutagenesis, selection, and enrichment into a single automated process, we achieved efficient and high-throughput evolution of CelB. The continuous culture system supported the evolution of a large variant library (~1.7×10⁹ evolving cells per culture) at any given time over the evolution experiment, enabling rapid optimization of CelB with minimal manual intervention.

### Screening and characterization of CelB variants

After completing the evolution experiment, the culture was screened using a blue-white screening method on Luria-Bertani (LB) agar plates containing X-gal and aTc. Dark-blue colonies, indicative of higher CelB activity, were selected and grown in LB medium with aTc to induce CelB expression. Following incubation with the inducer, cells were heated at 75°C for 15 min to obtain crude cell lysates, followed by the chlorophenol red-β-D-galactopyranoside (CPRG) assay in 96-well plates to confirm their β-galactosidase activity. This heat treatment served two purposes: it selected for thermostable CelB variants and disrupted the bacterial cell envelopes, allowing CPRG penetration for enzymatic activity assays.

**Fig 2:**
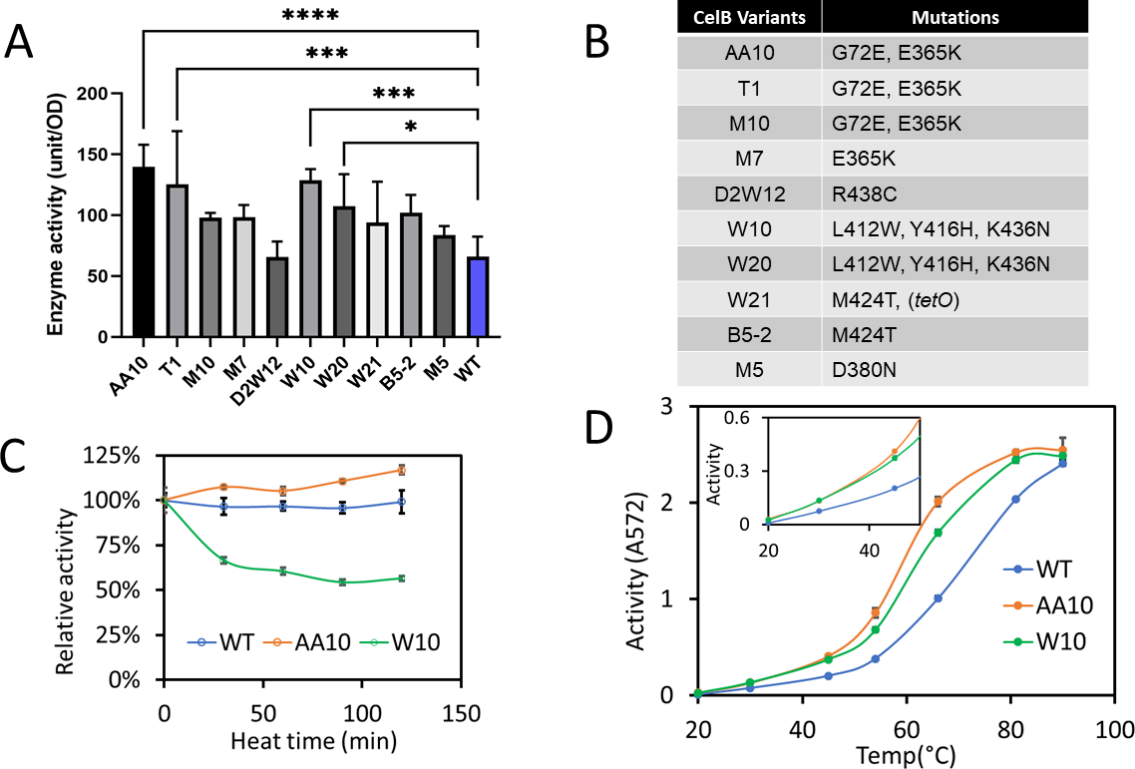
Characterization of CelB variants obtained from continuous directed evolution. (A) The CPRG assay results for CelB variants selected from blue-white screening. (B) Amino acid mutations in CelB variants, as identified by DNA sequencing. (C) Thermostability of CelB proteins was assessed using CPRG assay after heat treatment at 80°C over time. Enzymatic activities, represented by the slopes of CPR production curves, were measured at 25°C after each heat treatment and subsequently normalized to the initial activity measured without heat exposure. All proteins were overexpressed and purified before testing. (D) Enzymatic activity of CelB proteins across a range of temperatures. The inset provides a detailed view of activity levels below 50°C. Data are represented as means ± standard deviation of at least four replicates. Statistical significance: ^****^P <0.0001, ^***^P <0.001, ^*^P <0.05.

Ten variants showing relatively high activity in the CPRG assay are shown in Fig. 1 and 2. It is worth noting that not every colony selected from the blue-white screening exhibited significantly higher activity compared to the WT enzyme. This variability can be attributed to the preliminary nature of these screens, which are based on crude cell lysates and thus subject to experimental variability. Additionally, we observed that some mutants had similar activity to the WT, possibly due to a loss of thermal stability caused by heat treatment prior to the CPRG assay. While the CPRG assay itself was conducted at room temperature, the heat treatment step at 75°C may have compromised the stability of these variants. Compared to wild-type CelB, three variants including AA10, T1, and W10 exhibited a 70% increase in enzymatic activity in the heat-treated cells. The mutations of the ten were identified by DNA Sanger sequencing, and many of them shared the same mutations (Fig. 2B). Three variants, AA10, M10, and T1, share the identical mutations, including G72E and E365K, while M7 only acquired one mutation in E365K. Another two variants, W10 and W20, share the same mutations in L412W, Y416H, and K436N. B5-2 and W21 also shared the same mutation in M424T. There were no mutations found at the promoter region for all variants except W21 that acquired an additional mutation at the *tetO* operator that may transcriptionally increase protein expression.

We further characterized the activities of the two top-performing variants, AA10 and W10, under various conditions. To assess their thermostability, the purified proteins were heat-treated at 80°C for varying time intervals before testing their enzymatic activity at room temperature (Fig. 2C). While the wild-type enzyme maintained consistent activity, in agreement with previous findings (13–15), the two variants behaved differently. AA10 exhibited a slight increase in activity upon heat treatment, while W10 lost approximately 40% of its activity. Additionally, the activity of the variants was evaluated across a range of temperatures (Fig. 2D). We observed that, as the temperature increased from 20°C to 90°C, the activity of all enzymes rose rapidly. While wild-type CelB showed a steady increase in activity across this range, the two variants exhibited a faster rise in activity and reached a plateau around 80°C, which is lower than the wild-type enzyme’s optimum temperature of approximately 100°C (15). These results suggest that the GCCDE approach successfully shifted the optimal activity temperature of the two evolved enzymes. Although the two CelB variants showed similar activity to the wild type at 90°C, they displayed significantly higher activity at lower temperatures (30°C to 80°C). Our results demonstrate that the GCCDE approach effectively evolved and selected CelB variants that retained thermostability while exhibiting enhanced enzymatic activity at lower temperatures. Despite wild-type CelB being considered naturally highly optimized for stability and flexibility (15), our ability to engineer CelB variants with the desired performance in a single evolutionary experiment underscores the robustness and efficiency of this method. These findings highlight the potential of GCCDE to achieve protein engineering outcomes, even for proteins believed to be near their evolutionary limits.

### Kinetic study and protein structure analysis

**Fig 3:**
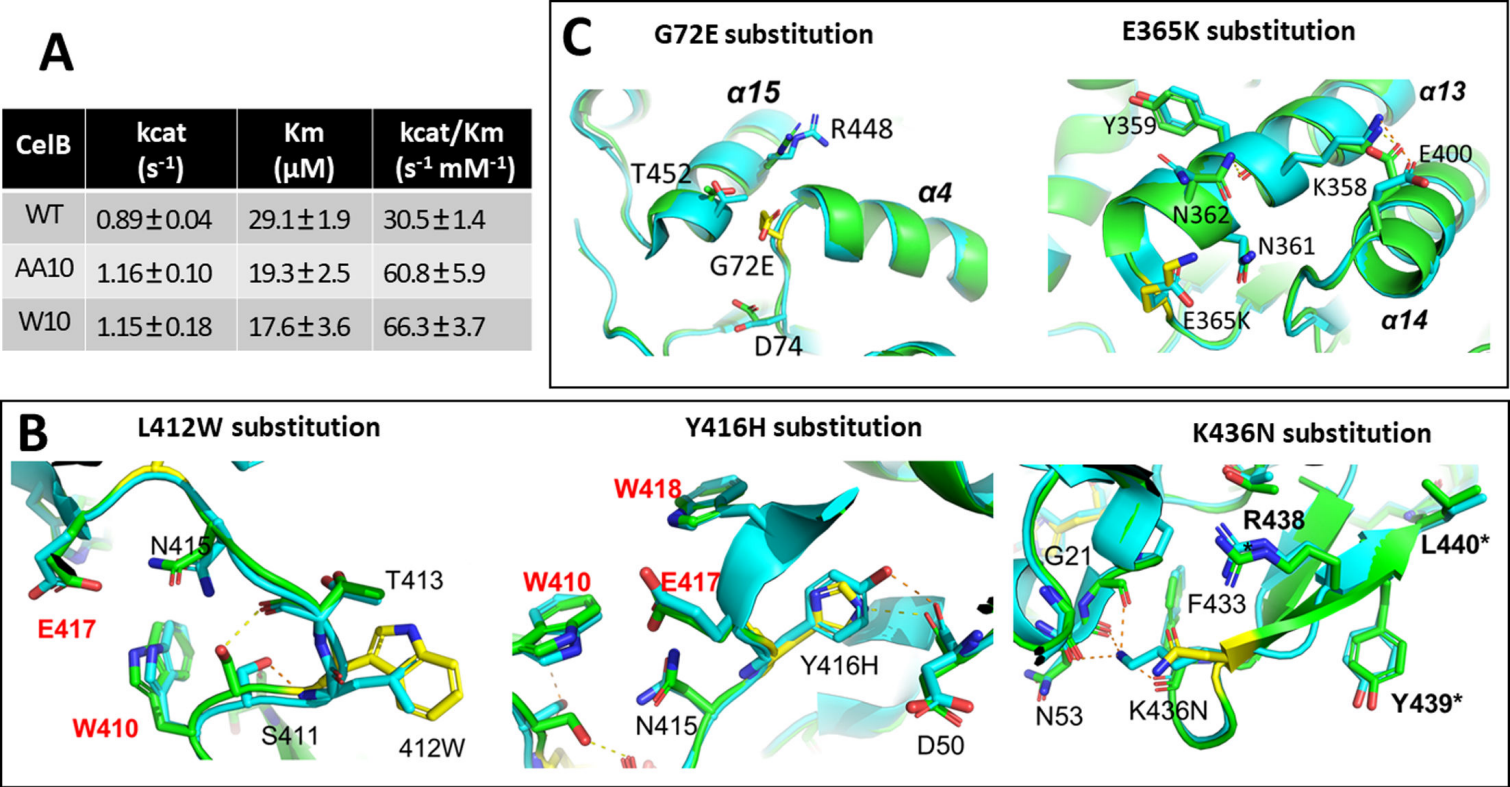
Kinetic parameters and structural analysis of CelB proteins. (A) The kinetic parameters of the wild-type CelB and two variants (AA10 and W10), measured at 25°C using CPRG as substrate. (B) Structural alignment of the wild type and W10. (C) Structural alignment of the wild type and AA10. The wild-type CelB (PDB id: 3APG) is in cyan, while variants are in green. The structures of variants were predicted from AlphaFold2. Residues in yellow color indicate amino acid substitutions. Active site residues are labeled in bolded red text, and inter-subunit residues are indicated by bolded text with an asterisk. Yellow and orange dashed lines indicate the hydrogen bonds formed between residues in the variants and in the wild type, respectively. The secondary structure elements, α-helices (⍺), are indicated in italics.

We next investigated the enzymatic kinetic parameters and mutations of AA10 and W10. The kinetic parameters of these variants were compared to those of the wild-type CelB (Fig. 3A). Both AA10 and W10 achieved an approximately 30% increase in *k*_cat_, the turnover number of the enzyme. Additionally, both mutants showed increased binding affinity toward the substrate, as indicated by lower *K*m values. Consequently, the overall catalytic efficiency (*k*_cat_/*K*m) for each variant increased twofold or more compared to the wild type at room temperature.

Amino acid substitutions in the two CelB variants are illustrated in the multiple sequence alignment with the wild-type protein (Fig. S1). The W10 variant harbors three key substitutions, L412W, Y416H, and K436N (Fig. 3B), that are located at the C-terminal region, which contains several well-conserved active-site residues and subunit-interface residues (15). To better explain the role of the mutations, the three-dimensional structure of W10 was predicted by AlphaFold2 (16) and compared to the structure of the wild-type protein that is publicly available (PDB id: 3APG). The substitution of L412 with a bulkier tryptophan near the active residue W410 likely facilitates substrate binding by reducing the size of the binding pocket. Additionally, the Y416H substitution could induce spatial shifts in the adjacent active residues including E417 and W418 (15), potentially promoting both galactose binding and catalytic efficiency of the enzyme (17). Lastly, K436N, located on the protein’s exterior near inter-subunit contact regions, may influence the protein’s overall thermostability. Replacing the charged lysine with an uncharged asparagine is predicted to disrupt the hydrogen bonds with G21, N53, and F433. This mutation also switches R438 and Y439, the residues for interfacial interactions and the homotetramer formation of CelB (15). Consequently, the K436N substitution may impair enzyme rigidity, which could explain the decreased stability of the W10 variant under heat treatment (Fig. 2C).

The AA10 variant contains two substitutions: G72E and E365K. Although the two mutations do not occur within the catalytic active sites, the resulting alterations in secondary structures could influence inter-subunit interactions that are critical for substrate entry and/or product release (18). These changes likely contribute to the enhanced catalytic activity and substrate binding affinity observed in the kinetic study (Fig. 3A). The glycine residue at position 72 is highly conserved across species (15), and replacing it with the charged glutamic acid introduces electrostatic attraction with R448 on the α15 helix (Fig. 3C). Additionally, the larger glutamic acid residue may introduce steric clashes to the local pocket, altering the arrangement of nearby residues, including T452 and D74. Consequently, the conformation of the α15 helix and the adjacent loop undergo significant rearrangements (Fig. 3B). The second substitution, E365K, reverses the charge at its position, which may induce conformational changes in the α13 and α14 helices. Collectively, these structural shifts in helices, including α13, α14, and α15, along with potential changes in neighboring helices, loops, and β-sheets, could significantly impact the overall architecture of the enzyme. These structural changes could enhance inter-subunit interactions, stabilize the quaternary structure, and promote the formation of homotetramers. Such rearrangements likely improve the cooperative dynamics and catalytic efficiency of CelB, ultimately enhancing its enzymatic performance.

### Conclusion

We have developed a growth-coupled continuous directed evolution approach that automates mutagenesis, selection, and enrichment simultaneously. Using this method, we successfully engineered the thermostable enzyme CelB to exhibit enhanced β-galactosidase activity at lower temperatures while maintaining its thermal stability, all within a single evolutionary experiment. This success demonstrates the effectiveness of our GCCDE approach, which enables genetic diversification, selection, and enrichment in a single process, significantly reducing time and effort required for PCR, cloning, and screening in traditional directed evolution. By integrating *in vitro* and *in vivo* mutagenesis, our method expands genetic diversity and improves evolutionary outcomes. Coupling enzymatic activity to cellular growth enables high-throughput selection of improved variants. With a continuous culture system and the MutaT7 platform, we achieved automated enzyme engineering with high-throughput and real-time selection of 1.7 × 10⁹ cells in real time in only one bioreactor. The resulting CelB variants showed expected higher β-galactosidase activity at room temperature while retaining their thermal stability. Key mutations were identified that likely improved substrate binding at lower temperatures while preserving overall structural integrity. This GCCDE approach is broadly applicable for engineering any protein whose activity can be linked to cellular growth, offering a powerful solution for high-throughput protein optimization and evolution.

## MATERIALS AND METHODS

### Strains and growth conditions

*E. coli* NEB 10-beta cells (NEB, cat# C3019H) were used for plasmid construction, and *E. coli* JM109 cells were utilized for protein overexpression in Luria-Bertani medium. NEB SOC outgrowth medium (NEB, cat# B9020S) was used for recovery following transformations. The *E. coli* Dual7 strain (10), derived from DH10B and lacking native β-galactosidase activity, was used for *in vivo* directed evolution experiments.

Lactose minimal medium, modified from M63 medium, contained lactose (10 g/L), (NH₄)₂SO₄ (2 g/L), KH₂PO₄ (20 g/L), K₂HPO₄ (48 g/L), FeSO₄ (0.5 mg/L), thiamine (0.5 mg/L), MgSO₄ (1 mM), NaCl (0.1 g/L), tryptone (0.2 g/L), and yeast extract (0.1 g/L), with pH adjusted to 7.0–7.2. M63-glucose medium was similarly prepared but substituted lactose with glucose (5 g/L). Kanamycin (50 µg/mL) was added when required.

### Plasmid construction and CelB library preparation

Plasmids were constructed using the NEBuilder HiFi DNA Assembly kit (NEB), and PCR reactions were performed with NEB Q5 High-Fidelity Polymerase. The *celB* gene from *Pyrococcus furiosus* (14) was synthesized by IDT and cloned into the pQE80 plasmid. The T5 promoter in pQE80 was modified by replacing one lacO sequence with tetO (5′-tccctatcagtgatagaga-3′) and mutating the second lacO to enable regulation solely by TetR and anhydrotetracycline (aTc). The CelB gene, engineered hybrid promoter (P_tetO), and TetR gene from pDA303 (19) were assembled to create plasmid pDA381. The low-copy-number pDA386 was then constructed from pDA381 by replacing its origin of replication with pSC101 Ori from pLC-F (20). Plasmid pDA386 served as the vehicle for *in vivo* mutagenesis and as the template for error-prone PCR. The plasmid maps and DNA sequences are provided in the Supplementary Materials. All the primers used to construct plasmids and the mutant library are shown in Table 1.

**TABLE 1:** Primers used in this study

Primer	Sequence 5′—3′	Notes
381-V1-F	ataatagattcaatccctatcagtgatagagatttcacacagaattcattaaagaggaga	*tetO* insertion
381-V1-R	aataatacgactcactatagggcttagc	pDA381 construction, error-prone PCR
381-V2-F	ttaatcactttacttttatctaatctggacacattcaccaccctgaattga	pDA381 construction
381-V2-R	ataatgtgtatccgactacaaagcaaataaattttttatgatttctcgaggtgaagacg	pDA381 construction
381-V3-F	gccctatagtgagtcgtattatttagctgagcttggactcct	pDA381 construction, CelB library
381-V3-R	tgaaagtgggtcctgagcgcaacgcaattaatgta	pDA381 construction
381-V4-F	cattaattgcgttgcgctcaggacccactttcacatttaagttg	pDA381 construction
381-V4-R	aatgtgtccagattagataaaagtaaagtgattaacagc	pDA381 construction
381overlap	tttatttgctttgtagtcggatacacattataatagattcaatccctatcagtgataga	T5 promoter modification
386-mut-F349	gatttcggctgggaaatgtatc	Error-prone PCR
386-bk-R	ggatacatttcccagccgaaatc	CelB starting library
386-R1	cttgttacagctcaacagtcacgacggtcacagcttgtctgt	pDA386 construction
386-F1	ttctacggggtctgacgctc	pDA386 construction
386-R2	tgagcgtcagaccccgtagaacgggtaagcctgttgatgatac	pDA386 construction
386-F2	gtgactgttgagctgtaacaag	pDA386 construction

Error-prone PCR was performed to generate a starting library targeting CelB’s catalytic region (amino acids 350–472) (13, 15, 17). The reaction mixture (40 µL) contained 0.6 ng/µL template DNA, 0.2 mM dATP/dGTP, 1 mM dCTP/dTTP, 5 mM MgCl_2_, 0.2 mM MnCl_2_, 0.05U/mL NEB Taq polymerase, and 0.4 µM primers (386-mut-F349 and 381-V1-R). PCR conditions were initial denaturation at 94°C for 2 min, 18 cycles of 94°C for 20 s, 50°C for 30 s, and 68°C for 30 s followed by a final extension at 68°C for 5 min. The resulting PCR product was verified by agarose gel electrophoresis, purified, and assembled into plasmids. These plasmids were transformed into *E. coli* Dual7 cells (10) using a previously established method (21), creating the CelB starting library. The library was recovered in 2 mL of SOC medium before inoculating to 15 mL of lactose minimal medium with kanamycin and aTc (20 ng/mL) for *in vivo* continuous evolution.

### Growth-coupling continuous directed evolution and selection

The starting CelB library culture prepared above (totally 17 mL) in a bioreactor was grown at 37°C in the Chi.Bio continuous culture system (22). When the OD_600_ reached 0.8, the turbidostat function was activated to keep this OD overnight by supplying fresh medium as needed. Subsequently, the OD_600_ was maintained at a constant level of 0.6 (~10⁸ CFU/mL, determined by viable counts on LB plates, mid-exponential phase) using turbidostatic setting in the Chi.Bio system, which automatically pumped in fresh medium as bacteria grew. The evolution experiment continued for 2 weeks, with approximately 1.7 × 10⁹ cells undergoing *in vivo* mutagenesis in the bioreactor at any given time.

Lactose in the medium activated MutaT7 expression, driving continuous mutagenesis of the *celB* gene downstream of the T7 promoter on the plasmids. Variants with higher enzymatic activity hydrolyzed lactose more efficiently, promoting faster bacterial growth. Slower-growing cells were gradually washed out of the bioreactor. The temperature was gradually reduced from 37°C to 27°C, selecting for CelB variants with enhanced β-galactosidase activity at lower temperatures. At the end of the evolution experiment, glucose was immediately added to the culture to a final concentration of 5 g/L to stop MutaT7 expression. The culture was then grown in M63-glucose medium within the same continuous system for 28 hours to stabilize mutations.

### Screening and measurement of β-galactosidase activity

The evolved cells were diluted and plated on LB agar plates supplemented with 5-bromo-4-chloro-3-indolyl-β-D-galactopyranoside (X-gal, 40 µg/mL), aTc (30 ng/mL), and kanamycin (50 µg/mL) for blue-white screening. Approximately 30 plates were screened, with each plate containing between 50 and 500 colonies depending on the dilution factor. The colonies exhibited a range of colors, from white to dark blue, indicating varying levels of activity. Dark blue colonies, indicative of high β-galactosidase activity, were selected and grown in LB medium with aTc induction to overexpress CelB. The cells were washed and resuspended in phosphate-buffered saline buffer (PBS, pH 7.4). The suspensions were heated at 75°C for 15 min to select heat-resistant variants and to lyse the cells, allowing CPRG to enter cells for enzymatic activity measurement. The crude cell lysate samples were then subjected to the CPRG assay.

In the CPRG assay, CelB hydrolyzes CPRG to release chlorophenol red (CPR), which was monitored at 572 nm (A_572_) using a microplate reader (Molecular Devices Inc.). CPRG was added to a final concentration of 0.5 mM in PBS buffer containing 2.7% glucose to screen for β-galactosidase variants tolerant to glucose inhibition. Enzymatic activity was calculated based on the slope of the CPR production curve, defined as an increase of 0.001 absorbance units per minute at A_572_. To compare enzymatic activity among variants, the density (OD_600_) of all cultures was measured to normalize enzymatic activity.

### Protein purification and characterization

The selected CelB variants were cloned into a high-copy-number plasmid with a 6×His tag using pDA381 as the backbone. Proteins were overexpressed in *E. coli* JM109 (DE3) upon aTc induction (30 ng/mL) and purified using Ni-NTA agarose resin (Thermo Fisher Scientific, cat# 88221), following the protocol described previously (19). Purity and molecular weight of the purified proteins were assessed by reducing SDS-PAGE analysis, and protein concentration was determined using a BCA assay (Thermo Fisher Scientific, cat# 23252). Enzymatic activity was measured using the CPRG assay described above. Kinetic parameters, including *Km* and *k_cat_*, were determined using the classical Eadie-Hofstee linearization plot (23). For thermostability testing, proteins were incubated at 80°C in a thermocycler (Bio-Rad Inc.) for various time intervals before conducting the CPRG assay at 25°C. Additionally, temperature-dependent activity was evaluated by incubating the purified enzymes with CPRG across varying temperatures for 15 min, followed by absorbance measurements at 572 nm (A_572_). Blank controls were prepared by incubating the CPRG solution without enzyme under the same conditions.

### Sequence alignment and structure analysis

Mutations in the selected high-activity variants were identified by DNA sequencing. The amino acid sequences of CelB variants were aligned with the wild-type CelB sequence using Clustal Omega (24) and rendered in EsPript 3 (25). Three-dimensional structural models of the CelB mutants were generated in the ColabFold platform (26) with AlphaFold2 (16). Structural visualization and alignment were conducted using PyMOL (version 2.5.4, Schrödinger, LLC).

### Statistics

All statistical analyses were performed in GraphPad Prism (version 10.2.3). One-way analysis of variance (ANOVA) followed by Tukey’s honest significant difference post-hoc tests were performed to compare mean values between variants and the wild type, with *P* <0.05 indicating statistical significance.

## Data Availability

All data generated during this study are included in this published article and its supplemental material. The GenBank accession numbers for the DNA sequences of the 10 CelB variants are PV246016 to PV246025.

## SUPPLEMENTAL MATERIAL

Supplemental material aem.02491-24-s0001.docx Figures S1 and S2 and plasmid DNA sequences.

ASM does not own the copyrights to Supplemental Material that may be linked to, or accessed through, an article. The authors have granted ASM a non-exclusive, world-wide license to publish the Supplemental Material files. Please contact the corresponding author directly for reuse.
