# Supplementary material for: Growth-coupled continuous directed evolution by MutaT7 enables efficient and automated enzyme engineering
Source: Appl Environ Microbiol. 2025 Mar 27;91(4):e02491-24. doi: 10.1128/aem.02491-24 (PMC12016552; doi:10.1128/aem.02491-24)
Supplement: Supplemental material — Figures S1 and S2 and plasmid DNA sequences. [file aem.02491-24-s0001.docx]

## **SUPPLEMENTAL MATERIAL**

**Growth-coupled continuous directed evolution by MutaT7 enables efficient and automated enzyme engineering**

Yijie Deng^1*^, Kai Etheridge^1^, Xinping Ran^2^, Hannah E. Maurais^1^, Rahul Sarpeshkar^1,3^

1. Thayer School of Engineering, Dartmouth College, Hanover, NH 03755, USA

2. Department of Biochemical Engineering, Duke University, Durham, 27708, USA

3. Departments of Engineering, Microbiology & Immunology, Physics, and

Molecular and Systems Biology, Dartmouth College, Hanover, NH 03755, USA

* Correspondence: [Yijie.deng@dartmouth.edu](mailto:Yijie.deng@dartmouth.edu)


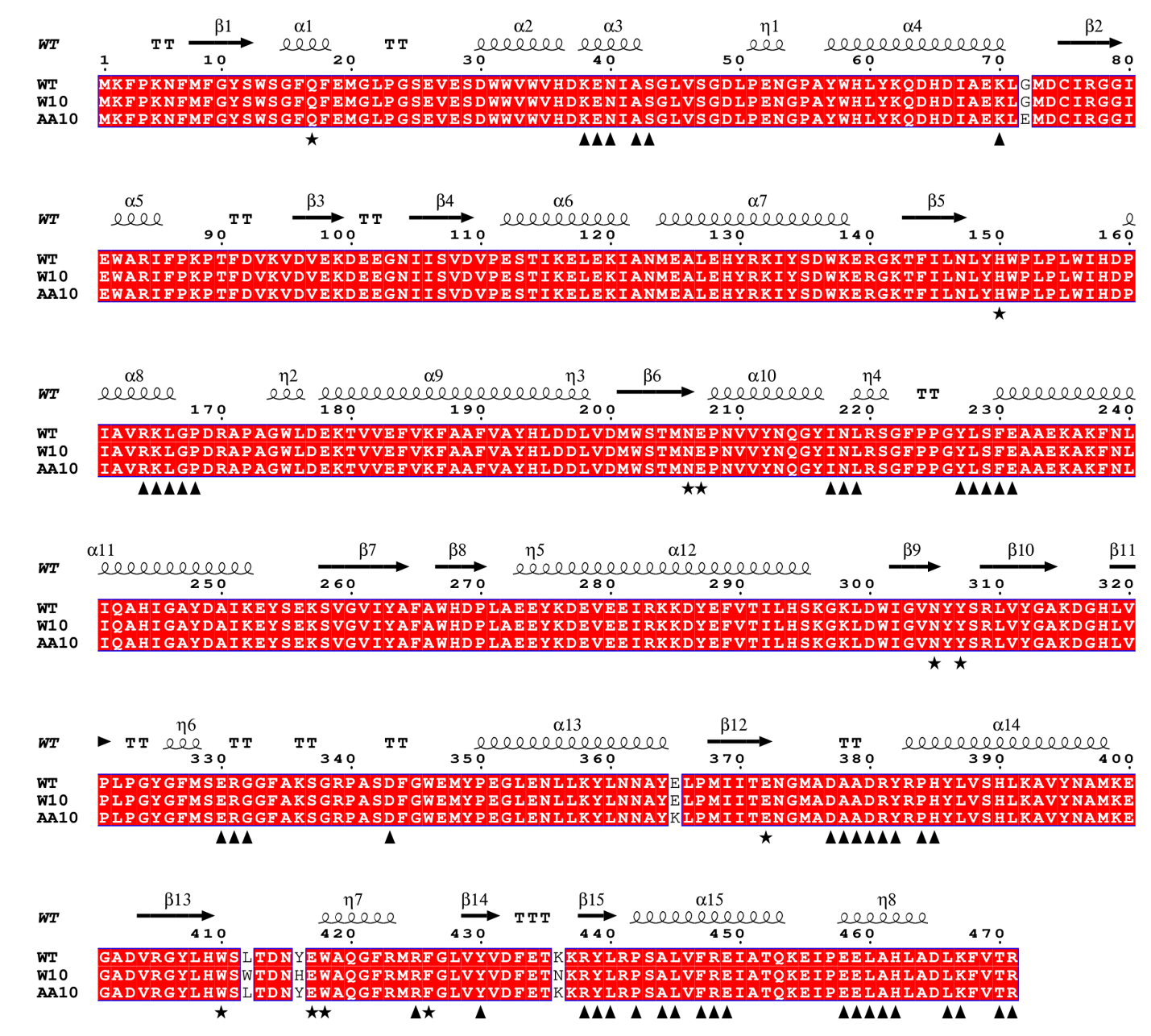


**Figure S1.** **Primary sequence alignment of wild-type CelB (PDB entry 3APG) with mutants W10 and AA10.** The secondary structure elements including beta sheets (β), alpha helices (⍺), 310 helices (η), and turns (TT) are shown above the alignment. Identical residues are depicted in white on a red background and mutated residues in black on a white background. The active site residues are marked with an asterisk below the sequences, while interfacial residues are indicated by triangles.


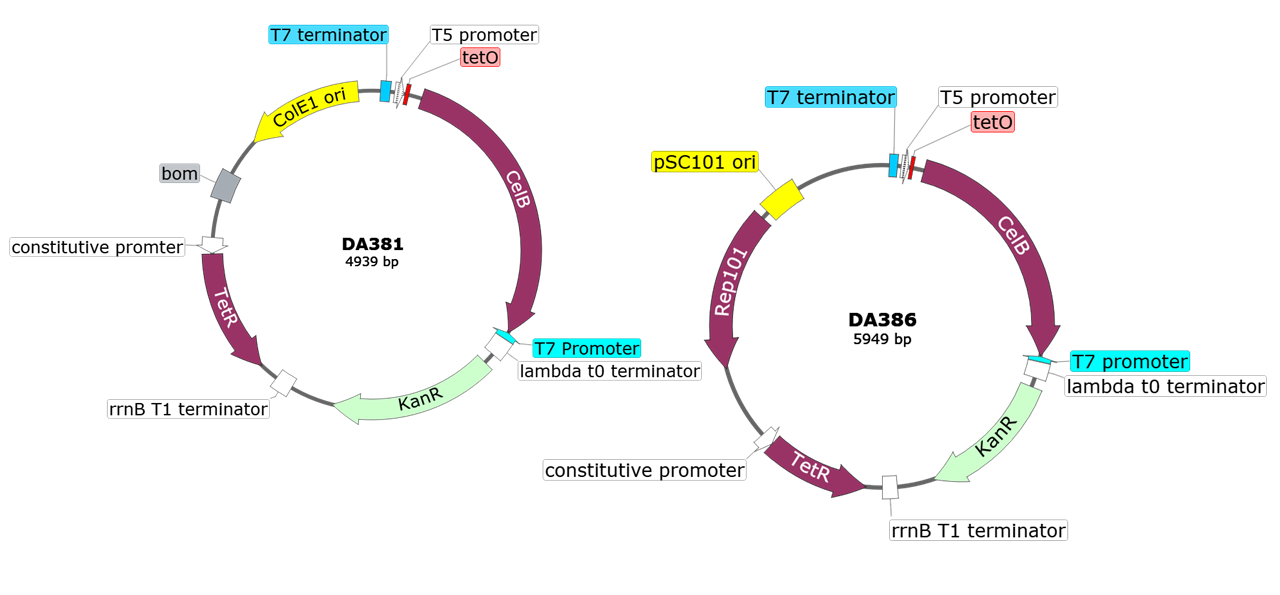


**Figure S2. Plasmid maps of pDA381 and pDA386 constructed in this study.** pDA381 is a high-copy-number plasmid carrying CelB gene while pDA386 is a low-copy-number plasmid carrying CelB gene. CelB: A tetrameric protein with both β-glucosidase and β-galactosidase activities;

T5: a constitutive promoter, which was modified to include *tetO* site make P_tetO hybrid promoter; ColE1: Origin of replication (Ori) for high-copy-number plasmid;

pSC101: Origin of replication for low-copy-number plasmid;

Rep101: protein required for replication of plasmids with pSC101 Ori;

T7: T7 promoter, used to drive MutaT7 proteins for *in vivo* mutagenesis;

TetR: A transcriptional repressor induced by aTc. TetR protein is constitutively expressed by its promoter; *tetO*: Tet operator, the binding site for TetR;

*rrnB* T1: a strong terminator; *KanR*: kanamycin resistant gene.

**DNA sequences for the plasmids constructed in this work**

**pDA381:**

TGGTCATGACATTAACCTATAAAAATAGGCGTATCACGAGGCCCAAAAAACCCCTCAAGACCCGTTTAGAGGCCCCAAGGGGTTATGCTAGCTTTCGTCTTCACCTCGAGAAATCATAAAAAATTTATTTGCTTTGTAGTCGGATACACATTATAATAGATTCAATCCCTATCAGTGATAGAGATTTCACACAGAATTCATTAAAGAGGAGAAATTAACCATGAGCGGCCATCACCATCATCATCACGGAAAGTTCCCGAAGAACTTTATGTTCGGTTACTCATGGTCTGGCTTTCAGTTTGAAATGGGTCTTCCCGGCAGCGAGGTCGAGAGTGATTGGTGGGTTTGGGTCCACGACAAAGAAAATATCGCTTCGGGGTTAGTGTCGGGGGACCTGCCAGAAAACGGTCCAGCCTATTGGCATCTTTACAAACAAGACCACGATATTGCAGAAAAGTTGGGAATGGACTGTATCCGTGGGGGGATCGAGTGGGCGCGCATCTTTCCGAAGCCGACGTTCGATGTTAAAGTGGATGTAGAGAAAGATGAAGAGGGGAATATCATCAGCGTAGATGTGCCAGAATCTACCATTAAAGAATTAGAAAAGATTGCGAACATGGAAGCCCTGGAGCATTATCGTAAAATCTACTCCGATTGGAAAGAGCGTGGCAAAACTTTCATCTTAAACTTATATCATTGGCCATTACCTCTTTGGATTCATGACCCGATCGCCGTTCGTAAGTTGGGCCCCGATCGCGCCCCCGCCGGATGGCTTGACGAAAAAACTGTAGTAGAGTTTGTTAAATTCGCCGCTTTTGTAGCATACCACCTTGACGACCTTGTTGACATGTGGTCCACAATGAACGAACCAAACGTGGTTTACAATCAAGGCTACATCAATTTGCGTTCTGGGTTTCCTCCTGGGTATTTGTCTTTCGAGGCTGCGGAAAAGGCCAAGTTCAATCTGATCCAGGCCCACATCGGCGCATACGACGCTATCAAAGAATACAGCGAAAAATCTGTCGGGGTAATCTATGCCTTTGCATGGCACGATCCCCTGGCCGAAGAGTACAAGGACGAAGTCGAAGAAATCCGCAAGAAGGACTACGAATTTGTGACAATTCTGCACTCGAAAGGCAAATTGGATTGGATCGGCGTCAATTATTACAGTCGCTTAGTATATGGCGCGAAGGACGGACATTTAGTGCCCTTACCAGGTTACGGGTTTATGTCGGAACGTGGTGGGTTCGCGAAATCTGGTCGCCCTGCGTCCGATTTCGGCTGGGAAATGTATCCTGAGGGGTTGGAAAACCTTTTAAAGTACCTTAACAATGCCTACGAGTTACCGATGATTATTACCGAAAACGGGATGGCTGATGCAGCGGACCGTTATCGCCCCCACTACCTTGTTTCACATCTGAAAGCCGTGTATAATGCAATGAAAGAGGGGGCCGACGTACGCGGTTACCTTCATTGGTCTTTGACTGATAATTACGAATGGGCTCAAGGATTCCGCATGCGCTTTGGGCTTGTCTACGTTGACTTCGAAACCAAAAAGCGCTACTTACGCCCCTCGGCGTTAGTTTTCCGCGAAATTGCGACCCAGAAAGAAATTCCTGAGGAACTGGCACACTTAGCAGACCTTAAATTTGTAACGCGCTAAGCCCTATAGTGAGTCGTATTATTTAGCTGAGCTTGGACTCCTGTTGATAGATCCAGTAATGACCTCAGAACTCCATCTGGATTTGTTCAGAACGCTCGGTTGCCGCCGGGCGTTTTTTATTGGTGAGAATCCAAGCTAGCCATGAAAATAAACTGTCTGCTTACATAAACAGTAATACAAGGGGTGTTATGAGCCATATTCAACGGGAAACGTCTTGCTCTAGGCCGCGATTAAATTCCAACATGGATGCTGATTTATATGGGTATAAATGGGCTCGCGATAATGTCGGGCAATCAGGTGCGACAATCTATCGATTGTATGGGAAGCCCGATGCGCCAGAGTTGTTTCTGAAACATGGCAAAGGTAGCGTTGCCAATGATGTTACAGATGAGATGGTCAGACTAAACTGGCTGACGGAATTTATGCCTCTTCCGACCATCAAGCATTTTATCCGTACTCCTGATGATGCTTGGTTACTCACGACTGCGATCCCCGGCAAAACAGCATTCCAGGTATTAGAAGAATATCCTGATTCAGGTGAAAATATTGTTGATGCGCTGGCAGTGTTCCTGCGCCGGTTGCATTCGATTCCTGTTTGTAATTGTCCTTTTAACAGCGATCGCGTATTTCGTCTCGCTCAGGCGCAATCACGAATGAATAACGGTTTGGTTGATGCGAGTGATTTTGATGACGAGCGTAATGGCTGGCCTGTTGAACAAGTCTGGAAAGAAATGCACAAACTTTTGCCATTCTCACCGGATTCAGTCGTCACTCATGGTGATTTCTCACTTGATAACCTTATTTTTGACGAGGGGAAATTAATAGGTTGTATTGATGTTGGACGAGTCGGAATCGCAGACCGATACCAGGATCTTGCCATCCTATGGAACTGCCTCGGTGAGTTTTCTCCTTCATTACAGAAACGGCTTTTTCAAAAATATGGTATTGATAATCCTGATATGAATAAATTGCAGTTTCATTTGATGCTCGATGAGTTTTTCTAAGAATTAATTCATGGGCAAATATTATACGCAAGGCGACAAGGTGCTGATGCCGCTGGCGATTCAGGTTCATCATGCCGTTTGTGATGGCTTCCATGTCGGCAGAATGCTTAATGAATTACAACAGTACTGCGATGAGTGGCAGGGCGGGGCGTAATTTTTTTAAGGCAGTTATTGGTGCCCTTAAACGCCTGGGGTAATGACTCTCTAGCTTGAGGCATCAAATAAAACGAAAGGCTCAGTCGAAAGACTGGGCCTTTCGTTTTATCTGTTGTTTGTCGGTGAACGCTCTCCTGAGTAGGACAAATCCGCCCTCTAGATTACGTGCAGTCGATGATAAGCTGTCAAACATGAGAATTGTGCCTAATGAGTGAGCTAACTTACATTAATTGCGTTGCGCTCAGGACCCACTTTCACATTTAAGTTGTTTTTCTAATCCGCATATGATCAATTCAAGGCCGAATAAGAAGGCTGGCTCTGCACCTTGGTGATCAAATAATTCGATAGCTTGTCGTAATAATGGCGGCATACTATCAGTAGTAGGTGTTTCCCTTTCTTCTTTAGCGACTTGATGCTCTTGATCTTCCAATACGCAACCTAAAGTAAAATGCCCCACAGCGCTGAGTGCATATAATGCATTCTCTAGTGAAAAACCTTGTTGGCATAAAAAGGCTAATTGATTTTCGAGAGTTTCATACTGTTTTTCTGTAGGCCGTGTACCTAAATGTACTTTTGCTCCATCGCGATGACTTAGTAAAGCACATCTAAAACTTTTAGCGTTATTACGTAAAAAATCTTGCCAGCTTTCCCCTTCTAAAGGGCAAAAGTGAGTATGGTGCCTATCTAACATCTCAATGGCTAAGGCGTCGAGCAAAGCCCGCTTATTTTTTACATGCCAATACAATGTAGGCTGCTCTACACCTAGCTTCTGGGCGAGTTTACGGGTTGTTAAACCTTCGATTCCGACCTCATTAAGCAGCTCTAATGCGCTGTTAATCACTTTACTTTTATCTAATCTGGACACATTCACCACCCTGAATTGACTCTCTTCCGGGCGCTATCATGCCATACCGCGAAAGGTTTTGCACCATTCGATGGTGTCGGAATTTCGGGCAGCGTTGGGTCCTGGCCACGGGTGCGCATGATCTAGAGCTGCCTCGCGCGTTTCGGTGATGACGGTGAAAACCTCTGACACATGCAGCTCCCGGCATCCGCTTACAGACAAGCTGTGACCGTCTCCGGGAGCAGACAAGCCCGTCAGGGCGCGTCAGCGGGTGTTGGCGGGTGTCGGGGCGCAGCCATGACCCAGTCACGTAGCGATAGCGGAGTGTATACTGGCTTAACTATGCGGCATCAGAGCAGATTGTACTGAGAGTGCACCATATGCGGTGTGAAATACCGCACAGATGCGTAAGGAGAAAATACCGCATCAGGCGCTCTTCCGCTTCCTCGCTCACTGACTCGCTGCGCTCGGTCGTTCGGCTGCGGCGAGCGGTATCAGCTCACTCAAAGGCGGTAATACGGTTATCCACAGAATCAGGGGATAACGCAGGAAAGAACATGTGAGCAAAAGGCCAGCAAAAGGCCAGGAACCGTAAAAAGGCCGCGTTGCTGGCGTTTTTCCATAGGCTCCGCCCCCCTGACGAGCATCACAAAAATCGACGCTCAAGTCAGAGGTGGCGAAACCCGACAGGACTATAAAGATACCAGGCGTTTCCCCCTGGAAGCTCCCTCGTGCGCTCTCCTGTTCCGACCCTGCCGCTTACCGGATACCTGTCCGCCTTTCTCCCTTCGGGAAGCGTGGCGCTTTCTCATAGCTCACGCTGTAGGTATCTCAGTTCGGTGTAGGTCGTTCGCTCCAAGCTGGGCTGTGTGCACGAACCCCCCGTTCAGCCCGACCGCTGCGCCTTATCCGGTAACTATCGTCTTGAGTCCAACCCGGTAAGACACGACTTATCGCCACTGGCAGCAGCCACTGGTAACAGGATTAGCAGAGCGAGGTATGTAGGCGGTGCTACAGAGTTCTTGAAGTGGTGGCCTAACTACGGCTACACTAGAAGGACAGTATTTGGTATCTGCGCTCTGCTGAAGCCAGTTACCTTCGGAAAAAGAGTTGGTAGCTCTTGATCCGGCAAACAAACCACCGCTGGTAGCGGTGGTTTTTTTGTTTGCAAGCAGCAGATTACGCGCAGAAAAAAAGGATCTCAAGAAGATCCTTTGATCTTTTCTACGGGGTCTGACGCTCAGTGGAACGAAAACTCACGTTAAGGGATTT

**pDA386:**

TGGTCATGACATTAACCTATAAAAATAGGCGTATCACGAGGCCCAAAAAACCCCTCAAGACCCGTTTAGAGGCCCCAAGGGGTTATGCTAGCTTTCGTCTTCACCTCGAGAAATCATAAAAAATTTATTTGCTTTGTAGTCGGATACACATTATAATAGATTCAATCCCTATCAGTGATAGAGATTTCACACAGAATTCATTAAAGAGGAGAAATTAACCATGAGCGGCCATCACCATCATCATCACGGAAAGTTCCCGAAGAACTTTATGTTCGGTTACTCATGGTCTGGCTTTCAGTTTGAAATGGGTCTTCCCGGCAGCGAGGTCGAGAGTGATTGGTGGGTTTGGGTCCACGACAAAGAAAATATCGCTTCGGGGTTAGTGTCGGGGGACCTGCCAGAAAACGGTCCAGCCTATTGGCATCTTTACAAACAAGACCACGATATTGCAGAAAAGTTGGGAATGGACTGTATCCGTGGGGGGATCGAGTGGGCGCGCATCTTTCCGAAGCCGACGTTCGATGTTAAAGTGGATGTAGAGAAAGATGAAGAGGGGAATATCATCAGCGTAGATGTGCCAGAATCTACCATTAAAGAATTAGAAAAGATTGCGAACATGGAAGCCCTGGAGCATTATCGTAAAATCTACTCCGATTGGAAAGAGCGTGGCAAAACTTTCATCTTAAACTTATATCATTGGCCATTACCTCTTTGGATTCATGACCCGATCGCCGTTCGTAAGTTGGGCCCCGATCGCGCCCCCGCCGGATGGCTTGACGAAAAAACTGTAGTAGAGTTTGTTAAATTCGCCGCTTTTGTAGCATACCACCTTGACGACCTTGTTGACATGTGGTCCACAATGAACGAACCAAACGTGGTTTACAATCAAGGCTACATCAATTTGCGTTCTGGGTTTCCTCCTGGGTATTTGTCTTTCGAGGCTGCGGAAAAGGCCAAGTTCAATCTGATCCAGGCCCACATCGGCGCATACGACGCTATCAAAGAATACAGCGAAAAATCTGTCGGGGTAATCTATGCCTTTGCATGGCACGATCCCCTGGCCGAAGAGTACAAGGACGAAGTCGAAGAAATCCGCAAGAAGGACTACGAATTTGTGACAATTCTGCACTCGAAAGGCAAATTGGATTGGATCGGCGTCAATTATTACAGTCGCTTAGTATATGGCGCGAAGGACGGACATTTAGTGCCCTTACCAGGTTACGGGTTTATGTCGGAACGTGGTGGGTTCGCGAAATCTGGTCGCCCTGCGTCCGATTTCGGCTGGGAAATGTATCCTGAGGGGTTGGAAAACCTTTTAAAGTACCTTAACAATGCCTACGAGTTACCGATGATTATTACCGAAAACGGGATGGCTGATGCAGCGGACCGTTATCGCCCCCACTACCTTGTTTCACATCTGAAAGCCGTGTATAATGCAATGAAAGAGGGGGCCGACGTACGCGGTTACCTTCATTGGTCTTTGACTGATAATTACGAATGGGCTCAAGGATTCCGCATGCGCTTTGGGCTTGTCTACGTTGACTTCGAAACCAAAAAGCGCTACTTACGCCCCTCGGCGTTAGTTTTCCGCGAAATTGCGACCCAGAAAGAAATTCCTGAGGAACTGGCACACTTAGCAGACCTTAAATTTGTAACGCGCTAAGCCCTATAGTGAGTCGTATTATTTAGCTGAGCTTGGACTCCTGTTGATAGATCCAGTAATGACCTCAGAACTCCATCTGGATTTGTTCAGAACGCTCGGTTGCCGCCGGGCGTTTTTTATTGGTGAGAATCCAAGCTAGCCATGAAAATAAACTGTCTGCTTACATAAACAGTAATACAAGGGGTGTTATGAGCCATATTCAACGGGAAACGTCTTGCTCTAGGCCGCGATTAAATTCCAACATGGATGCTGATTTATATGGGTATAAATGGGCTCGCGATAATGTCGGGCAATCAGGTGCGACAATCTATCGATTGTATGGGAAGCCCGATGCGCCAGAGTTGTTTCTGAAACATGGCAAAGGTAGCGTTGCCAATGATGTTACAGATGAGATGGTCAGACTAAACTGGCTGACGGAATTTATGCCTCTTCCGACCATCAAGCATTTTATCCGTACTCCTGATGATGCTTGGTTACTCACGACTGCGATCCCCGGCAAAACAGCATTCCAGGTATTAGAAGAATATCCTGATTCAGGTGAAAATATTGTTGATGCGCTGGCAGTGTTCCTGCGCCGGTTGCATTCGATTCCTGTTTGTAATTGTCCTTTTAACAGCGATCGCGTATTTCGTCTCGCTCAGGCGCAATCACGAATGAATAACGGTTTGGTTGATGCGAGTGATTTTGATGACGAGCGTAATGGCTGGCCTGTTGAACAAGTCTGGAAAGAAATGCACAAACTTTTGCCATTCTCACCGGATTCAGTCGTCACTCATGGTGATTTCTCACTTGATAACCTTATTTTTGACGAGGGGAAATTAATAGGTTGTATTGATGTTGGACGAGTCGGAATCGCAGACCGATACCAGGATCTTGCCATCCTATGGAACTGCCTCGGTGAGTTTTCTCCTTCATTACAGAAACGGCTTTTTCAAAAATATGGTATTGATAATCCTGATATGAATAAATTGCAGTTTCATTTGATGCTCGATGAGTTTTTCTAAGAATTAATTCATGGGCAAATATTATACGCAAGGCGACAAGGTGCTGATGCCGCTGGCGATTCAGGTTCATCATGCCGTTTGTGATGGCTTCCATGTCGGCAGAATGCTTAATGAATTACAACAGTACTGCGATGAGTGGCAGGGCGGGGCGTAATTTTTTTAAGGCAGTTATTGGTGCCCTTAAACGCCTGGGGTAATGACTCTCTAGCTTGAGGCATCAAATAAAACGAAAGGCTCAGTCGAAAGACTGGGCCTTTCGTTTTATCTGTTGTTTGTCGGTGAACGCTCTCCTGAGTAGGACAAATCCGCCCTCTAGATTACGTGCAGTCGATGATAAGCTGTCAAACATGAGAATTGTGCCTAATGAGTGAGCTAACTTACATTAATTGCGTTGCGCTCAGGACCCACTTTCACATTTAAGTTGTTTTTCTAATCCGCATATGATCAATTCAAGGCCGAATAAGAAGGCTGGCTCTGCACCTTGGTGATCAAATAATTCGATAGCTTGTCGTAATAATGGCGGCATACTATCAGTAGTAGGTGTTTCCCTTTCTTCTTTAGCGACTTGATGCTCTTGATCTTCCAATACGCAACCTAAAGTAAAATGCCCCACAGCGCTGAGTGCATATAATGCATTCTCTAGTGAAAAACCTTGTTGGCATAAAAAGGCTAATTGATTTTCGAGAGTTTCATACTGTTTTTCTGTAGGCCGTGTACCTAAATGTACTTTTGCTCCATCGCGATGACTTAGTAAAGCACATCTAAAACTTTTAGCGTTATTACGTAAAAAATCTTGCCAGCTTTCCCCTTCTAAAGGGCAAAAGTGAGTATGGTGCCTATCTAACATCTCAATGGCTAAGGCGTCGAGCAAAGCCCGCTTATTTTTTACATGCCAATACAATGTAGGCTGCTCTACACCTAGCTTCTGGGCGAGTTTACGGGTTGTTAAACCTTCGATTCCGACCTCATTAAGCAGCTCTAATGCGCTGTTAATCACTTTACTTTTATCTAATCTGGACACATTCACCACCCTGAATTGACTCTCTTCCGGGCGCTATCATGCCATACCGCGAAAGGTTTTGCACCATTCGATGGTGTCGGAATTTCGGGCAGCGTTGGGTCCTGGCCACGGGTGCGCATGATCTAGAGCTGCCTCGCGCGTTTCGGTGATGACGGTGAAAACCTCTGACACATGCAGCTCCCGGCATCCGCTTACAGACAAGCTGTGACCGTCGTGACTGTTGAGCTGTAACAAGTTGTCTCAGGTGTTCAATTTCATGTTCTAGTTGCTTTGTTTTACTGGTTTCACCTGTTCTATTAGGTGTTACATGCTGTTCATCTGTTACATTGTCGATCTGTTCATGGTGAACAGCTTTAAATGCACCAAAAACTCGTAAAAGCTCTGATGTATCTATCTTTTTTACACCGTTTTCATCTGTGCATATGGACAGTTTTCCCTTTGATATCTAACGGTGAACAGTTGTTCTACTTTTGTTTGTTAGTCTTGATGCTTCACTGATAGATACAAGAGCCATAAGAACCTCAGATCCTTCCGTATTTAGCCAGTATGTTCTCTAGTGTGGTTCGTTGTTTTTGCGTGAGCCATGAGAACGAACCATTGAGATCATGCTTACTTTGCATGTCACTCAAAAATTTTGCCTCAAAACTGGTGAGCTGAATTTTTGCAGTTAAAGCATCGTGTAGTGTTTTTCTTAGTCCGTTACGTAGGTAGGAATCTGATGTAATGGTTGTTGGTATTTTGTCACCATTCATTTTTATCTGGTTGTTCTCAAGTTCGGTTACGAGATCCATTTGTCTATCTAGTTCAACTTGGAAAATCAACGTATCAGTCGGGCGGCCTCGCTTATCAACCACCAATTTCATATTGCTGTAAGTGTTTAAATCTTTACTTATTGGTTTCAAAACCCATTGGTTAAGCCTTTTAAACTCATGGTAGTTATTTTCAAGCATTAACATGAACTTAAATTCATCAAGGCTAATCTCTATATTTGCCTTGTGAGTTTTCTTTTGTGTTAGTTCTTTTAATAACCACTCATAAATCCTCATAGAGTATTTGTTTTCAAAAGACTTAACATGTTCCAGATTATATTTTATGAATTTTTTTAACTGGAAAAGATAAGGCAATATCTCTTCACTAAAAACTAATTCTAATTTTTCGCTTGAGAACTTGGCATAGTTTGTCCACTGGAAAATCTCAAAGCCTTTAACCAAAGGATTCCTGATTTCCACAGTTCTCGTCATCAGCTCTCTGGTTGCTTTAGCTAATACACCATAAGCATTTTCCCTACTGATGTTCATCATCTGAGCGTATTGGTTATAAGTGAACGATACCGTCCGTTCTTTCCTTGTAGGGTTTTCAATCGTGGGGTTGAGTAGTGCCACACAGCATAAAATTAGCTTGGTTTCATGCTCCGTTAAGTCATAGCGACTAATCGCTAGTTCATTTGCTTTGAAAACAACTAATTCAGACATACATCTCAATTGGTCTAGGTGATTTTAATCACTATACCAATTGAGATGGGCTAGTCAATGATAATTACTAGTCCTTTTCCTTTGAGTTGTGGGTATCTGTAAATTCTGCTAGACCTTTGCTGGAAAACTTGTAAATTCTGCTAGACCCTCTGTAAATTCCGCTAGACCTTTGTGTGTTTTTTTTGTTTATATTCAAGTGGTTATAATTTATAGAATAAAGAAAGAATAAAAAAAGATAAAAAGAATAGATCCCAGCCCTGTGTATAACTCACTACTTTAGTCAGTTCCGCAGTATTACAAAAGGATGTCGCAAACGCTGTTTGCTCCTCTACAAAACAGACCTTAAAACCCTAAAGGCTTAAGTAGCACCCTCGCAAGCTCGGGCAAATCGCTGAATATTCCTTTTGTCTCCGACCATCAGGCACCTGAGTCGCTGTCTTTTTCGTGACATTCAGTTCGCTGCGCTCACGGCTCTGGCAGTGAATGGGGGTAAATGGCACTACAGGCGCCTTTTATGGATTCATGCAAGGAAACTACCCATAATACAAGAAAAGCCCGTCACGGGCTTCTCAGGGCGTTTTATGGCGGGTCTGCTATGTGGTGCTATCTGACTTTTTGCTGTTCAGCAGTTCCTGCCCTCTGATTTTCCAGTCTGACCACTTCGGATTATCCCGTGACAGGTCATTCAGACTGGCTAATGCACCCAGTAAGGCAGCGGTATCATCAACAGGCTTACCCGTTCTACGGGGTCTGACGCTCAGTGGAACGAAAACTCACGTTAAGGGATTT
